# Two methoxy derivatives of resveratrol, 3,3′,4,5′-tetramethoxy-*trans*-stilbene and 3,4′,5-trimethoxy-*trans*-stilbene, suppress lipopolysaccharide-induced inflammation through inactivation of MAPK and NF-κB pathways in RAW 264.7 cells

**DOI:** 10.1186/s13020-021-00480-9

**Published:** 2021-08-04

**Authors:** Chunxiu Zhou, Xutao Zhang, Cheng-Chao Ruan, Wai San Cheang

**Affiliations:** 1 State Key Laboratory of Quality Research in Chinese Medicine, Institute of Chinese Medical Sciences, University of Macau, Room 5008a, Building N22, Avenida da Universidade, Taipa, Macao, SAR China; 2 Department of Physiology and Pathophysiology, School of Basic Medical Sciences, Shanghai Key Laboratory of Bioactive Small Molecules, Fudan University, Shanghai, China

**Keywords:** 3,3′,4,5′-tetramethoxy-*trans*-stilbene, 3,4′,5-trimethoxy-*trans*-stilbene, Inflammation, Lipopolysaccharide, MAPK, NF-κB

## Abstract

**Background:**

3,3′,4,5′-tetramethoxy-*trans*-stilbene (3,3′,4,5′-TMS) and 3,4′,5-trimethoxy-*trans*-stilbene (3,4′,5-TMS) are two methoxy derivatives of resveratrol. Previous researches have proved that resveratrol and its analogues have anti-inflammatory effect through suppressing mitogen-activated protein kinase (MAPK) and nuclear factor-κB (NF-κB) signaling pathways. This study aims to study whether 3,3′,4,5′-TMS and 3,4′,5-TMS alleviate inflammation and the underlying mechanism.

**Methods:**

RAW 264.7 macrophage cells were treated with lipopolysaccharide (LPS) to induce inflammation and pretreated with 3,3′,4,5′-TMS or 3,4′,5-TMS. Cell viability was measured with the 3-(4,5)-dimethylthiazol-2-yl)-2,5-diphenyltetrazolium bromide (MTT) assay. Nitric oxide (NO) release was detected by Griess reagent. The secretions of pro-inflammatory cytokines were assessed by ELISA kits. Protein expressions of signaling molecules were determined by Western blotting. Reactive oxygen species (ROS) production was detected by fluorescence staining and malondialdehyde (MDA) assay.

**Results:**

3,3′,4,5′-TMS and 3,4′,5-TMS suppressed LPS-induced NO release and pro-inflammatory cytokines (IL-6 and TNF-α) secretions in a dose-dependent manner in RAW 264.7 cells. 3,3′,4,5′-TMS and 3,4′,5-TMS significantly down-regulated the LPS-induced expressions of cyclooxygenase-2 (COX-2) and inducible nitric oxide synthase (iNOS), and partially suppressed the activation of MAPK (phosphorylation of p38, JNK, ERK), and NF-κB (phosphorylation of IKKα/β, p65 and IκBα) signaling pathways; where phosphorylation of ERK and p65 was mildly but not significantly decreased by 3,3′,4,5′-TMS. LPS-induced NF-κB/p65 nuclear translocation was inhibited by both 3,3′,4,5′-TMS and 3,4′,5-TMS. Moreover, both resveratrol derivatives decreased the ROS levels.

**Conclusions:**

3,3′,4,5′-TMS and 3,4′,5-TMS significantly suppress LPS-induced inflammation in RAW 264.7 cells through inhibition of MAPK and NF-κB signaling pathways and also provide anti-oxidative effect. This study reveals potential therapeutic applications of 3,3′,4,5′-TMS and 3,4′,5-TMS for inflammatory diseases.

## Background

Inflammation, widely defined as a nonspecific response to tissue injury or infection, is a complicated and pervasive form of defense and is employed by both innate and adaptive immune systems to combat pathogenic intruders [1]. Macrophage plays a critical role in the initiation, maintenance, and resolution of inflammation and it can be activated by many signals including cytokines [interferon γ (IFN-γ), tumor necrosis factor α (TNF-α), and granulocyte-monocyte colony stimulating factor (GM-CSF)], bacterial lipopolysaccharide (LPS), extracellular matrix proteins, and other chemical mediators [2]. When inflammation is triggered by a pathogen, resident macrophages produce various pro-inflammatory mediators such as nitric oxide (NO) from inducible nitric oxide synthase (iNOS), cyclooxygenase-2 (COX-2), reactive oxygen species (ROS), TNF-α, and interleukin (IL)-6 [2, 3].

Recognition of LPS by toll-like receptor 4 (TLR4) consequently activates the downstream signaling cascades, including the activation of mitogen-activated protein kinases (MAPKs) and nuclear factor kappa-light-chain-enhancer of activated B cells (NF-κB) family [4]. The family members of MAPKs mainly include c-Jun N-terminal kinase/stress-activated protein kinase (JNK/SAPK), extracellular signal-regulated kinase (ERK), and p38 mitogen-activated protein kinase (p38 MAPK) [5, 6]. NF-κB family consists of p50, p52, RelA (p65), RelB, c-Rel, NF-κB1 (p105), and NF-κB2 (p100) [7]. Moreover, phosphorylation of inhibitor of NF-κB (IκB) kinase (IKK) complex leads to phosphorylation-induced IκB degradation, releasing NF-κB subunit from the cytoplasm to the nucleus to induce the transcription of pro-inflammatory genes [6]. Therefore, inhibition of such excessive inflammatory cytokines and related mediators through suppressing the activation of MAPKs and NF-κB can be an important target for drug discovery and development of treatment against inflammation.

**Fig. 1:**
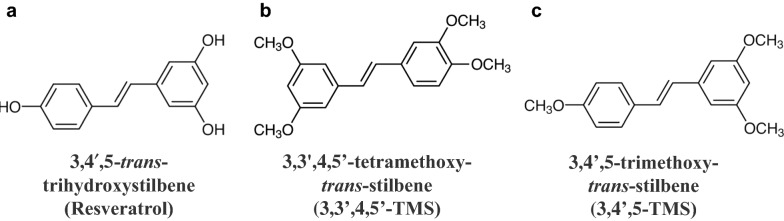
Chemical structures of **a** 3,4′,5-trans-trihydroxystilbene (resveratrol), **b** 3,3′,4,5′-tetramethoxy-trans-stilbene (3,3′,4,5′-TMS), and **c** 3,4′,5-trimethoxy-trans-stilbene (3,4′,5-TMS)

Resveratrol (3,4′,5-*trans*-trihydroxystilbene; Fig. 1a) is a hydroxylated derivative of stilbene and is also a natural phytoalexin found in grape skins and seeds, peanuts, red wine, blueberries and so on [8]. Resveratrol was originally considered as a potential anti-aging agent. It has later been proved by many basic science investigations and more than 240 clinical trials to exert many pharmacological activities against chronic diseases, including inflammatory diseases, diabetes mellitus, hypertension, cardiovascular diseases (CVDs), Alzheimer’s disease, kidney diseases, liver diseases, and cancers [9–11]. Despite these remarkable health benefits, low bioavailability of resveratrol limits its application in effective treatments [12–15]. As a result, researchers have paid more attention on structural modification of resveratrol. According to the structure-activity relationship (SAR) studies, the parent structure of resveratrol is vital for its therapeutic effects [16]. In recent years, more and more natural or synthetic resveratrol derivatives have been studied especially the methoxylated, hydroxylated, and halogenated ones that exert diverse therapeutic potential [8]. 3,3′,4,5′-tetramethoxy-*trans*-stilbene (3,3′,4,5′-TMS; Fig. 1b) and 3,4′,5-trimethoxy-*trans*-stilbene (3,4′,5-TMS; Fig. 1c) are two methoxy derivatives of resveratrol. Some previous studies have revealed some potent therapeutic effects of 3,4′,5-TMS, inhibiting inflammation in macrophages, breast cancer cells invasiveness and high glucose-induced endothelial dysfunction [17–19].

To date, only few studies explore the anti-inflammatory effect of 3,4′,5-TMS and even no studies working on 3,3′,4,5′-TMS. In the present study, we used LPS-induced RAW 264.7 macrophages as an in vitro model to investigate the anti-inflammatory effects of 3,3′,4,5′-TMS and 3,4′,5-TMS and the underlying mechanism. The potency was also compared.

## Methods

### Chemicals and reagents

3,3′,4,5′-TMS and 3,4′,5-TMS (purity > 98%) were purchased from Tokyo Chemical Industry Co., Ltd. High-glucose Dulbecco’s modified Eagle’s medium (DMEM) were obtained from GE Healthcare Life Sciences HyClone Laboratories (Utah, USA) while fetal bovine serum (FBS) and Penicillin-streptomycin (P/S) were from Gibco (Carlsbad, CA, USA). LPS, 3-(4,5-dimethylthiazol-2-yl)-2,5-diphenyltetrazolium bromide (MTT), Griess reagent, bovine serum albumin (BSA) and Triton X-100 were purchased from Sigma-Aldrich (St. Louis, MO, USA). Enzyme-linked immunosorbent assay (ELISA) kits for IL-6 and TNF-α were purchased from Mlbio (Shanghai, China). All the primary antibodies were purchased from Cell Signaling Tech (Danvers, MA, USA) while the secondary antibodies were obtained from Beyotime Biotechnology (Shanghai, China). 4% paraformaldehyde (PFA), Alexa Fluor 488-labeled secondary antibody, and 2-(4-amidinophenyl)-6-indolecarbamidine dihydrochloride (DAPI) were purchased from Beyotime Biotechnology (Shanghai, China). Dihydroethidium (DHE) and 5-(and-6)-chloromethyl-2′,7′-dichlorodihydrofluorescein diacetate acetyl ester (CM-H_2_DCFDA) were acquired from Molecular Probes (Eugene, OR, USA). Malondialdehyde (MDA) assay kit was purchased from Nanjing Jiancheng Bioengineering Institute (Nanjing, China).

### Cell culture

RAW 264.7, a mouse macrophage cell line from the American Type Culture Collection (ATCC, Manassas, VA, USA), was cultured in high-glucose DMEM supplemented with 10% FBS plus 100 U/mL penicillin and 100 µg/mL streptomycin at 37 ℃ in a humidified incubator with 5% CO_2_.

### Cell viability analysis

The effects of 3,3′,4,5′-TMS and 3,4′,5-TMS on the cell viability were determined by MTT assay. RAW 264.7 cells were seeded into 96-well plates (7 × 10^3^ cells/well, 100 µL medium/well). After incubating overnight, the cells were treated with 3,3′,4,5′-TMS and 3,4′,5-TMS at final concentrations of 0, 5, 10, 30 and 50 µM for another 24 h. The 3,3′,4,5′-TMS and 3,4′,5-TMS were dissolved in DMEM medium. Thereafter, the MTT reaction solution (20 µL, 5 mg/mL) was added into each well, followed by 3 h of incubation at 37 ℃. At last, the supernatants were removed and the formazan crystals in each well were dissolved in 150 µL of DMSO. After being shaken for 30 min, the 96-well plates were read by the SpectraMax M5 microplate reader (Molecular Devices, Silicon Valley, CA, United States) at 570 nm to detect the absorbance.

### Determination of nitric oxide (NO) release

The RAW 264.7 cells were seeded in 24-well plates (2 × 10^5^ cells/well) and allowed to adhere overnight at 37 ℃ with 5% CO_2_. The cells were pretreated with 3,3′,4,5′-TMS (10 and 50 µM) or 3,4′,5-TMS (10 and 50 µM) for 4 h and then co-treated with LPS (1 µg/mL) for another 12 h. The release of NO was determined by measuring the accumulated nitrite in the culture medium with Griess reagent according to the manufacturer’s instructions. The absorbance was detected at 548 nm using the microplate reader.

### Enzyme-linked immunosorbent assay (ELISA)

RAW 264.7 cells (6 × 10^5^ cells/well in 6-well plates) were pretreated with different concentrations (10 and 50 µM) of 3,3′,4,5′-TMS or 3,4′,5-TMS for 4 h, followed by the co-incubation of LPS (1 µg/mL) for 12 h. The culture media were collected after the drug treatment and the releases of IL-6 and TNF-α from the cells were detected using immunoassay kits (Mlbio, Shanghai, China) according to the manufacturer’s protocol. The absorbance values at 450 nm were determined by the SpectraMax M5 microplate reader.

### Western blotting analysis

RAW 264.7 cells (6 × 10^5^ cells/well in 6-well plates) were pretreated with of 3,3′,4,5′-TMS or 3,4′,5-TMS at 10 and 50 µM for 4 h, followed by the addition of LPS (1 µg/mL) for 12 h. The cells were harvested on ice and lysed with RIPA solution containing 1% Protease Inhibitor Cocktail (Beyotime Biotechnology, Shanghai, China) and 1% phenylmethanesulfonyl fluoride (PMSF). The cell lysates were centrifuged at 15,000 rpm for 30 min at 4 ℃ to collect supernatants. The total protein content was determined by BCA assay (Beyotime Biotechnology, Shanghai, China). Protein samples were separated by 8 or 10% SDS-PAGE gels and the blots were transferred to polyvinylidene fluoride (PVDF) membranes (Millipore, Billerica, MA, USA). The membrane was blocked by 5% skimmed milk or 1% BSA in Tween-20 phosphate-buffered saline (TBST) buffer, followed by incubation with appropriate primary antibodies overnight at 4 ℃ and with the corresponding secondary antibodies for another 1 h at room temperature. Finally, the specific protein bands were visualized with an American ECL™ Advanced Western Blotting Detection Kit (GE Healthcare Life Sciences, Uppsala, Sweden) and scanned by ChemiDoc™ MP Imaging System (BIO-RAD, USA).

### Immunofluorescence assay

RAW 264.7 cells (3 × 10^5^ cells/confocal dish) were pretreated with 3,3′,4,5′-TMS or 3,4′,5-TMS (50 µM) for 4 h, followed by the co-incubation of LPS (1 µg/mL) for 12 h. The cells were then washed by PBS for 10 min and fixed with 4% PFA at room temperature for 20 min. The fixed cells were permeabilized with 0.1% Triton X-100 for 10 min and blocked with 3% BSA for 1 h at room temperature. Then the cells were incubated with the primary NF-κB p65 antibody (1:500) overnight at 4 ℃. Finally, the cells were washed by PBS for 10 min and were incubated with Alexa Fluor 488-labeled secondary antibody (1:100) for 1 h at 37 ℃. After washed, the cells nuclei were dyed by DAPI. The fluorescence images were captured under the Leica-DMi8 Inverted fluorescence microscope (Leica, Wetzlar, Germany).

### Fluorescence imaging

RAW 264.7 cells (1.5 × 10^5^ cells/well in 24-well plates) were pre-treated with different concentrations (10 and 50 µM) of 3,3′,4,5′-TMS or 3,4′,5-TMS for 4 h and LPS (1 µg/mL) was added for another 4 h. Cells were stained with DHE or CM-H_2_DCFDA to specifically detect the generation of intracellular superoxide radical (O_2_^•−^) and hydrogen peroxide (H_2_O_2_), respectively. Cells were incubated with 5 µmol/L DHE/ CM-H_2_DCFDA-containing normal physiological saline solution (NPSS) in dark for 30 min at 37 ℃ in NPSS contained (mM): 140 NaCl, 5 KCl, 1 CaCl2, 1 MgCl2, 10 glucose, and 5 HEPES (pH 7.4). The fluorescence levels of DHE and CM-H_2_DCFDA were determined by the fluorescence shift using a flow cytometer (BD LSR Fortessa cytometry) and Leica-DMi8 Inverted fluorescent microscope respectively. Densitometry analysis was performed by the Image-Pro Plus 6.0 software.

### MDA assay

RAW 264.7 cells (6 × 10^5^ cells/well in 6-well plates) were pretreated with of 3,3′,4,5′-TMS or 3,4′,5-TMS at 10 and 50 µM for 4 h, followed by the addition of LPS (1 µg/mL) for 12 h. The cells were harvested on ice and lysed with RIPA solution containing 1% Protease Inhibitor Cocktail and 1% PMSF. The cell lysates were centrifuged at 15,000 rpm for 30 min at 4 ℃ to collect supernatants. The total protein content was determined by BCA assay. The MDA content was then detected with assay kit according to the manufacturer’s instructions. All the results were normalized by the total protein content.

### Statistical analysis

All data of experiments are showed as mean ± standard error of mean (SEM) of n independent experiments. Variance between two groups was analyzed using one-way analysis of variance (ANOVA) by GraphPad Prism software (GraphPad Software, United States). *P* < 0.05 is considered to be statistically significant.

## Results

### 3,3′4,5′-TMS and 3,4′,5-TMS reverse the effects of LPS on NO release and morphology in RAW 264.7 cells without affecting cell viability

**Fig. 2:**
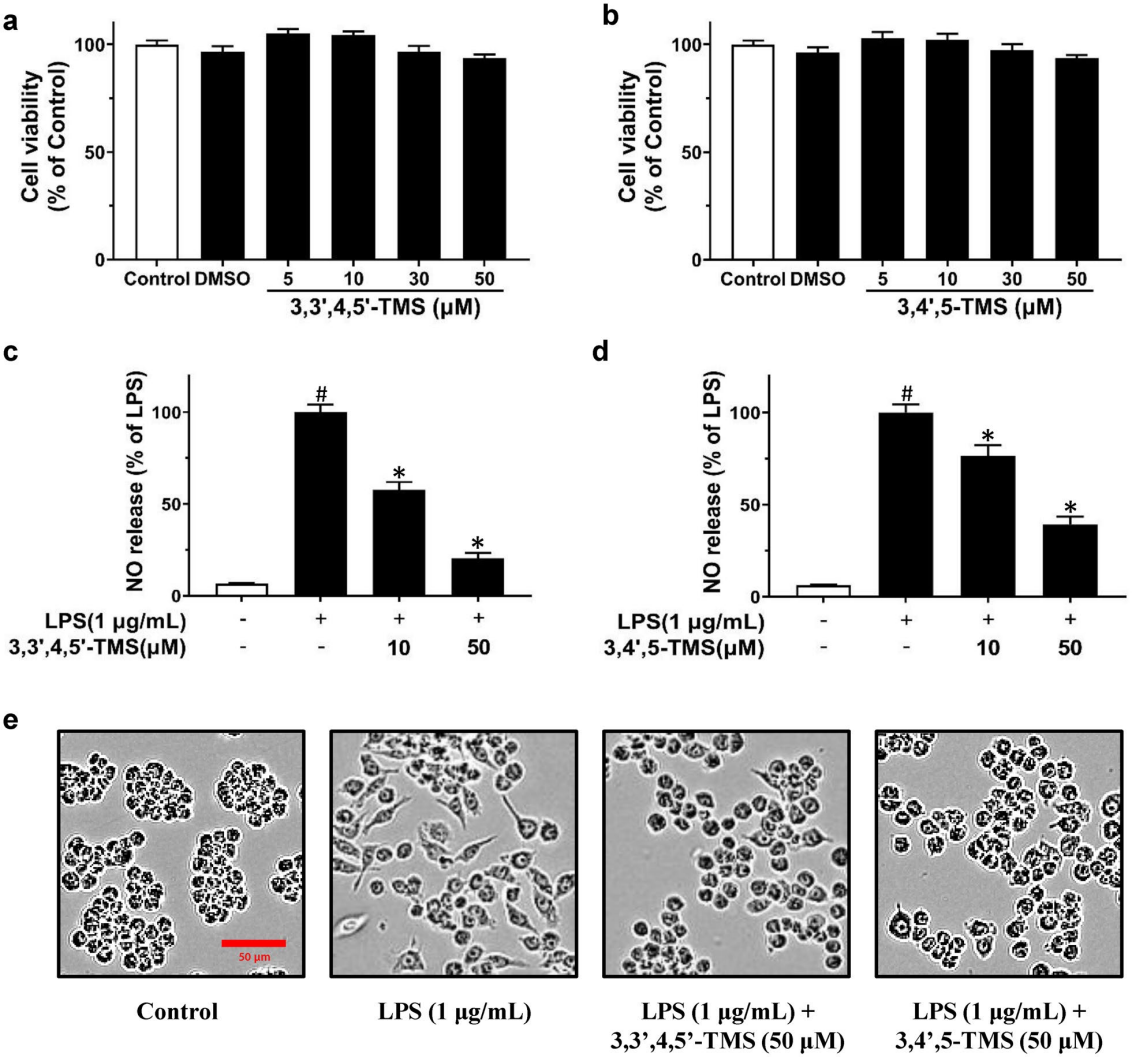
Effects of 3,3′4,5′-TMS and 3,4′,5-TMS on cell viability, NO release and morphology in RAW 264.7 cells. **a**,** b** Cell viability of RAW 264.7 cells treated with different concentrations (5, 10, 30, 50 µM) of 3,3′4,5′-TMS and 3,4′,5-TMS for 24 h. **c**, **d** NO release in RAW 264.7 cells pretreated with 3,3′,4,5′-TMS or 3,4′,5-TMS (10 and 50 µM) for 4 h and then co-treated with lipopolysaccharides (LPS, 1 µg/mL) for another 12 h. Data are expressed as the mean ± SEM, n = 3. ^#^*P* < 0.05 vs control. *P < 0.05 vs LPS. **e** Morphology of RAW 264.7 cells from different groups was visualized by optical microscopy (×10)

MTT assay was performed to ensure the effects of 3,3′,4,5′-TMS and 3,4′,5-TMS on cell viability. RAW 264.7 cells were treated with 3,3′,4,5′-TMS and 3,4′,5-TMS at different concentrations (0, 5, 10, 30 and 50 µM) for 24 h. The results showed that 3,3′,4,5′-TMS and 3,4′,5-TMS had no significant effect on the cell viability on RAW 264.7 cells (Fig. 2a, b). Therefore, we explored the effects of 3,3′,4,5′-TMS and 3,4′,5-TMS on RAW 264.7 cells treated with 1 µg/mL LPS at the safe concentrations of 10 and 50 µM.

To investigate whether 3,3′,4,5′-TMS and 3,4′,5-TMS have anti-inflammatory properties, NO release levels were measured in cell culture supernatants. The results showed that the NO release was significantly enhanced upon LPS stimulation (1 µg/mL, 12 h) while 3,3′,4,5′-TMS and 3,4′,5-TMS suppressed the NO release in LPS-induced cells in a dose-dependent manner (Fig. 2c, d). The inhibitory rates of NO release by 3,3′,4,5′-TMS at 10 and 50 µM were 42 and 80% respectively; whereas those for 3,4′,5-TMS were 24 and 61% respectively.

As shown in Fig. 2e, morphological changes in RAW 264.7 cells were obvious. LPS-stimulated RAW 264.7 cells differentiated into an irregular shape with pseudopodia and accelerated spreading as compared to the normal state in the control group. When treated by 3,3’,4,5’-TMS and 3,4’,5-TMS in addition, the cells showed less pseudopodia formation and cell spreading.

### 3,3′4,5′-TMS and 3,4′,5-TMS inhibit LPS-induced production of cytokines IL-6 and TNF-α in RAW 264.7 cells

**Fig. 3:**
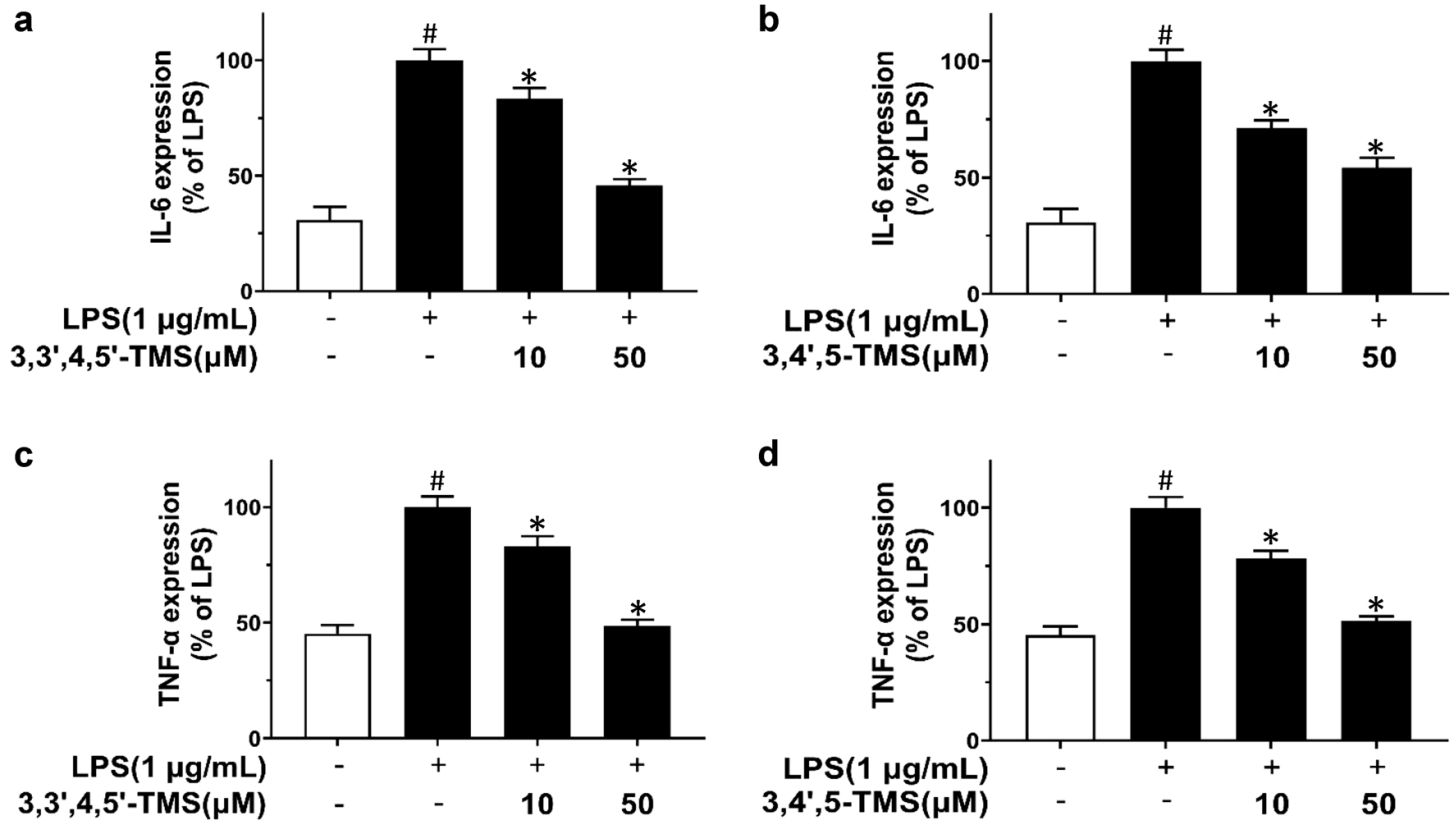
Effects of 3,3′4,5′-TMS and 3,4′,5-TMS on **a**, **b** IL-6 and **c**, **d** TNF-α secretions in LPS-stimulated RAW 264.7 cells. The cells were pretreated with 3,3′,4,5′-TMS or 3,4′,5-TMS (10 and 50 µM) for 4 h followed by the addition of LPS (1 µg/mL) for 12 h. Data are expressed as the mean ± SEM, n = 4. ^#^*P* < 0.05 vs control. *P < 0.05 vs LPS

ELISA was used to access the production of pro-inflammatory cytokines. LPS stimulation for 12 h significantly enhanced IL-6 (Fig. 3a, b) and TNF-α levels (Fig. 3c, d) in RAW 264.7 cells. Pretreatment of 3,3′,4,5′-TMS (10, 50 µM) and 3,4′,5-TMS (10, 50 µM) for 4 h before addition of LPS (1 µg/mL, 12 h) attenuated the production of these two cytokines in a dose-dependent manner. The inhibitory rates of IL-6 by 3,3′,4,5′-TMS were 17 and 54% respectively; whilst those for 3,4′,5-TMS were 29 and 46% respectively. Similar inhibitory effects were found for TNF-α generation: 17% at 10 µM and 52% at 50 µM of 3,3′,4,5′-TMS, and also 22% at 10 µM and 49% at 50 µM of 3,4′,5-TMS.

### 3,3′4,5′-TMS and 3,4′,5-TMS upregulate the expression of inflammatory markers iNOS and COX-2

**Fig. 4:**
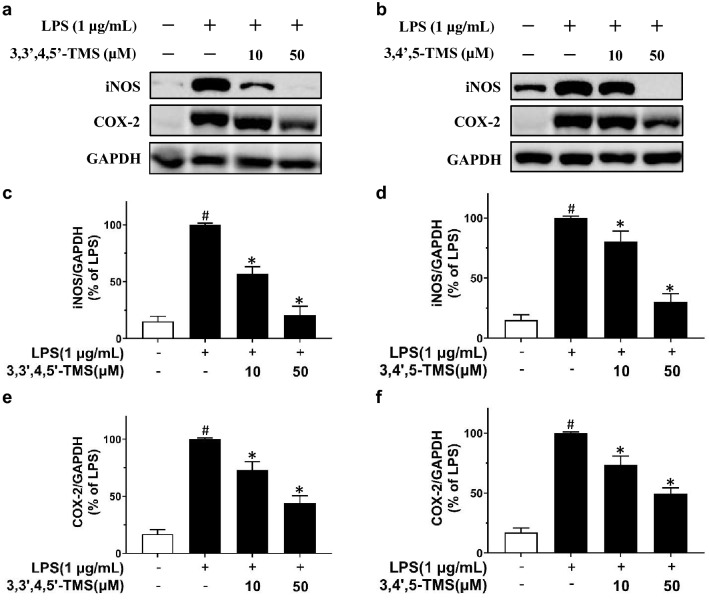
Effects of 3,3′4,5′-TMS and 3,4′,5-TMS on expression levels of iNOS and COX-2 in RAW 264.7 macrophages. **a**, **b** Representative blots and summarized data showing the effects of 3,3’4,5’-TMS and 3,4’,5-TMS pretreated for 4 h upon LPS stimulation (1 µg/mL, 12 h) on **c**, **d** iNOS and **e**, **f** COX-2 expressions. Data are expressed as the mean ± SEM, n = 3–4. ^#^*P* < 0.05 vs control. *P < 0.05 vs LPS

As measured by western blotting Protein expression of iNOS (Fig. 4a–d) and COX-2 (Fig. 4a, b, e, f) normalized to GAPDH was increased by LPS stimulation and notably decreased by 3,3′,4,5′-TMS (10, 50 µM) and 3,4′,5-TMS (10, 50 µM) treatment in RAW 264.7 cells through a dose-dependent manner.

### 3,3′4,5′-TMS and 3,4′,5-TMS exert anti-inflammatory effects by suppressing LPS-induced MAPK signaling pathway in RAW 264.7 cells

**Fig. 5:**
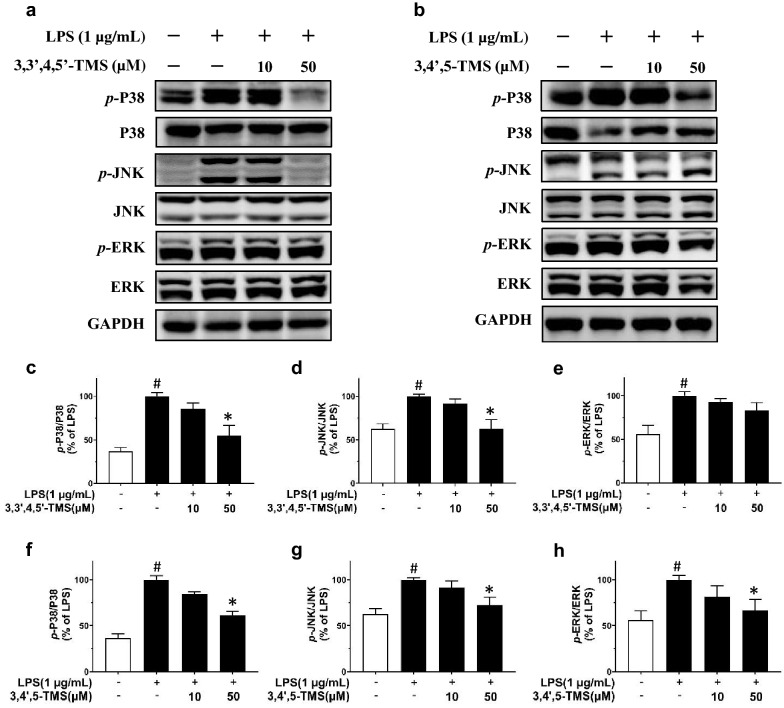
Effects of 3,3′4,5′-TMS and 3,4′,5-TMS on MAPK signaling pathway. The cells were pretreated with 3,3′,4,5′-TMS or 3,4’,5-TMS (10 and 50 µM) for 4 h and then co-treated with LPS (1 µg/mL) for 12 h. **a**, **b** Representative blots and summarized data showing the effects of **c**–**e** 3,3′4,5′-TMS and **f**–**h** 3,4′,5-TMS on the phosphorylation of P38 at Thr180/Tyr182, JNK at Thr183/Tyr185, and ERK at Thr202/Tyr204 normalized to its respective total protein. Data are expressed as the mean ± SEM, n = 3–4. ^#^*P* < 0.05 vs control. *P < 0.05 vs LPS

Three members of MAPK signaling pathway were detected. Phosphorylation levels of p38 at Thr180/Tyr182, JNK at Thr183/Tyr185, and ERK at Thr202/Tyr204 normalized to its respective total protein was all upregulated by LPS stimulation for 12 h (Fig. 5). After 4 h pretreatment of 3,3′,4,5′-TMS and co-treatment with LPS (1 µg/mL, 12 h), phosphorylation of p38 and JNK was effectively suppressed at the concentration of 50 µM (Fig. 4a, c, d). Besides, Western blot of cell lysate showed small but not significant downregulation of phosphorylated ERK (Fig. 4a, e). Likewise, the expressions of *p*-p38, *p*-JNK, and *p*-ERK were all significantly suppressed by 3,4′,5-TMS treatment at the concentration of 50 µM (Fig. 4b, f–h).

### 3,3′4,5′-TMS and 3,4′,5-TMS inhibit NF-κB signaling pathway in RAW 264.7 cells

**Fig. 6:**
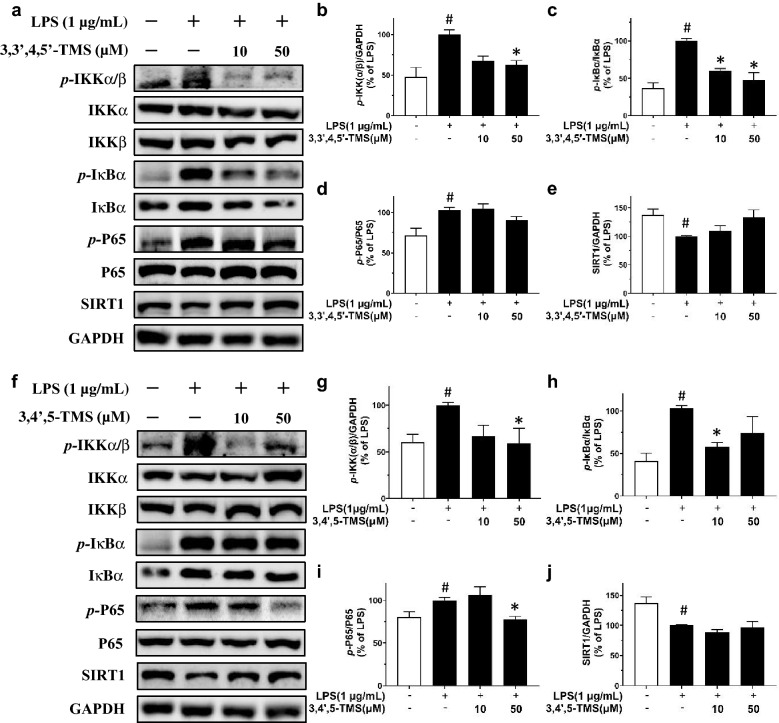
Effects of 3,3′4,5′-TMS and 3,4′,5-TMS on NF-κB signaling pathway. The cells were pretreated with 3,3′,4,5′-TMS or 3,4′,5-TMS (10 and 50 µM) followed by LPS incubation (1 µg/mL) for 12 h. **a** Representative blots and summarized data showing the effect of 3,3′4,5′-TMS on the phosphorylation of **b** IKKα/β at Ser176/180, **c** IκBα at Ser32, and **d** P65 at Ser536, as well as **e** SIRT1 expression as compared to its respective total protein or GAPDH. **f** Representative blots and **g–j** summarized data showing the effects of 3,4′,5-TMS. Data are expressed as the mean ± SEM, n = 3–4. ^#^*P* < 0.05 vs control. *P < 0.05 vs LPS

**Fig. 7:**
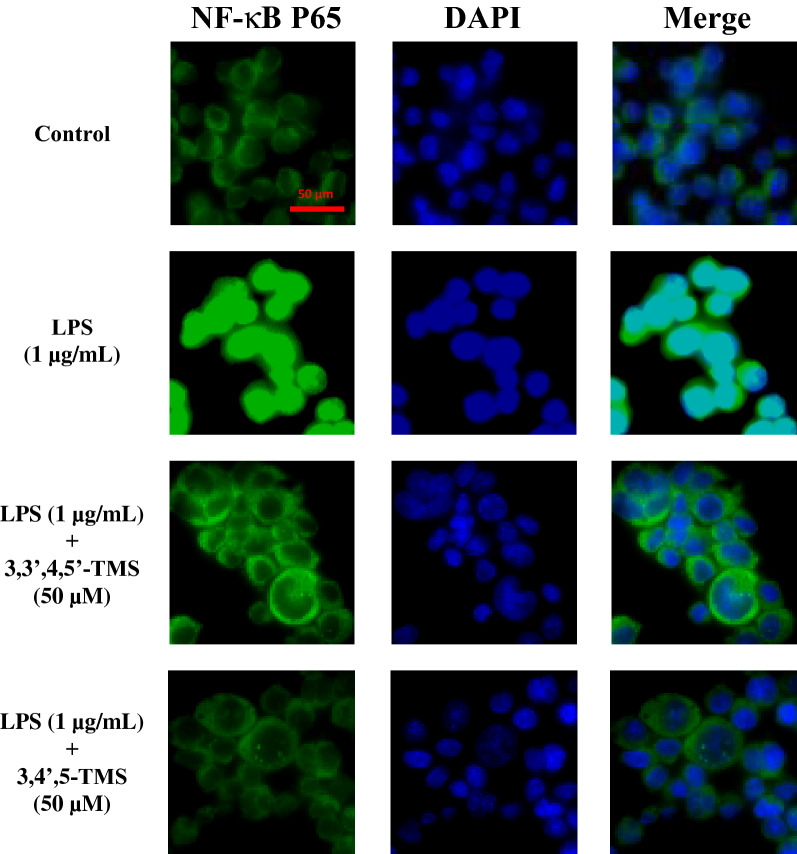
Effects of 3,3′4,5′-TMS and 3,4′,5-TMS on NF-κB p65 nuclear translocation. The cells were pretreated with 3,3′,4,5′-TMS or 3,4′,5-TMS (50 µM) for 4 h and then co-treated with LPS (1 µg/mL) for another 4 h for immunofluorescence staining. P65 protein was represented by green fluorescence while the nuclear was indicated by blue fluorescence

LPS treatment (1 µg/mL, 12 h) activated NF-κB signaling pathway, increasing *p*-IKKα/β at Ser176/180, *p*-IκBα at Ser32, and *p*-p65 at Ser536 as well as downregulating SIRT1 expression in RAW 264.7 cells (Fig. 6). Pretreatment of 3,3′,4,5′-TMS down-regulated p-IKKα/β and p-IκBα without any effect on the total protein expressions of IKKα, IKKβ or IκBα (Fig. 6a–c). However, 3,3′,4,5′-TMS showed minor but not significant reversal on p-P65 and SIRT1 expression (Fig. 6a, d, e). For 3,4′,5-TMS, after 4 h pretreatment and co-treatment with LPS (1 µg/mL, 12 h), all LPS-induced phosphorylation of IKKα/β, IκBα and p65 was significantly decreased but SIRT1 expression was unaltered (Fig. 6f–j). Importantly, LPS treatment (1 µg/mL, 4 h) led to NF-κB p65 nuclear translocation; and such translocation was prevented by pretreatment of 3,3′,4,5′-TMS and 3,4′,5-TMS (50 µM, 4 h) (Fig. 7). The immunofluorescence results showed the inhibitory effect of 3,3′,4,5′-TMS and 3,4′,5-TMS on the nuclear translocation of NF-κB p65.

### 3,3′4,5′-TMS and 3,4′,5-TMS diminishes LPS-induced oxidative stress in RAW 264.7 cells

**Fig. 8:**
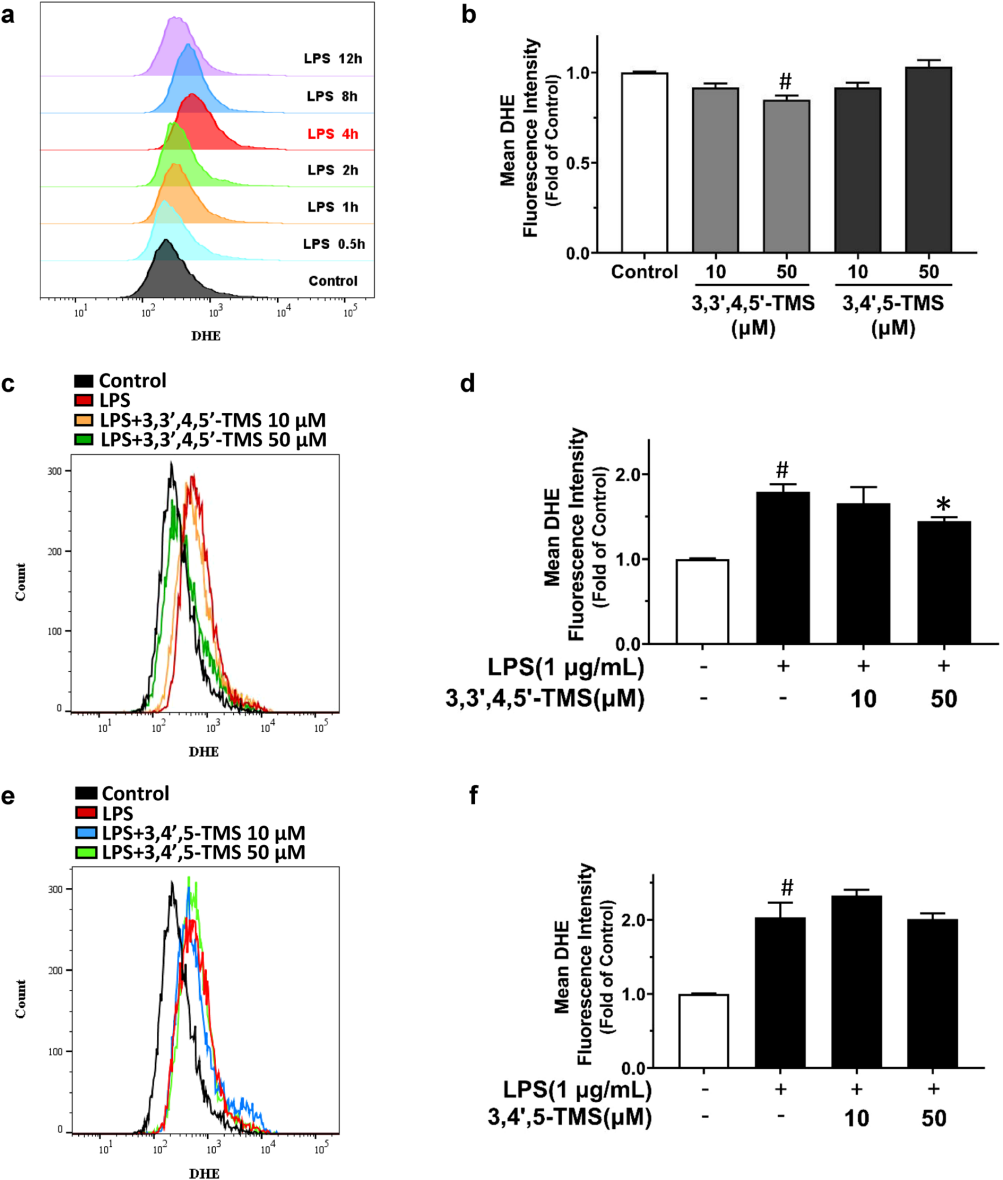
Effects of 3,3′4,5′-TMS and 3,4′,5-TMS superoxide production in RAW 264.7 cells. **a** The cells were treated with LPS (1 µg/mL) for 0.5 h, 1 h, 2 h, 4 h, 8 h, and 12 h. **b** The cells were treated with 3,3′,4,5′-TMS or 3,4′,5-TMS (10 and 50 µM) for 4 h in the absence of LPS. The cells were pretreated with 3,3′,4,5′-TMS or 3,4′,5-TMS (10 and 50 µM) for 4 h and then added with LPS (1 µg/mL) for another 4 h. Representative fluorescence shit and summarized data showing the levels of superoxide production determined using DHE staining with flow cytometry in response to **c**, **d** 3,3′4,5′-TMS and **e**, **f** 3,4′,5-TMS treatment in LPS-treated cells. Data are expressed as the mean ± SEM, n = 4. ^#^*P* < 0.05 vs control. *P < 0.05 vs LPS

**Fig. 9:**
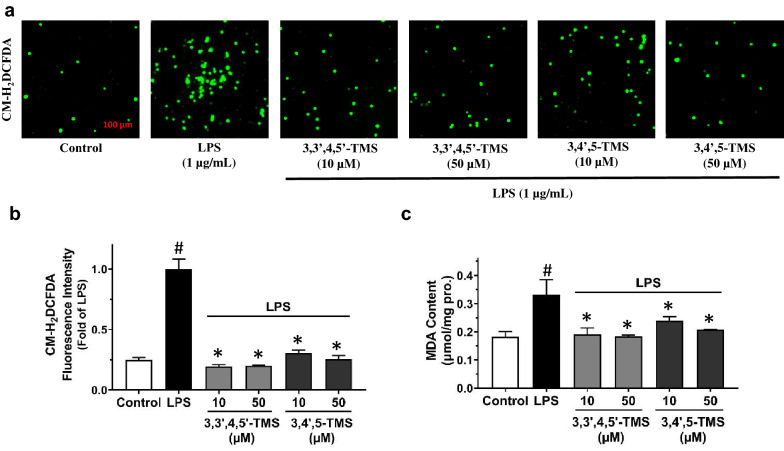
Effects of 3,3′4,5′-TMS and 3,4′,5-TMS on CM-H_2_DCFDA intensity and MDA content in RAW 264.7 cells. **a** Representative images and **b** summarized data showing CM-H_2_DCFDA intensity in cells pretreated with 3,3′,4,5′-TMS or 3,4′,5-TMS (10 and 50 µM) for 4 h and co-treated with LPS (1 µg/mL) for another 4 h. **c** MDA content was determined in the cells pretreated with 3,3′,4,5′-TMS (50 µM) or 3,4′,5-TMS (50 µM) for 4 h and co-treated with LPS (1 µg/mL) for another 12 h with normalization to the total protein content. Data are expressed as the mean ± SEM, n = 4. ^#^*P* < 0.05 vs control. *P < 0.05 vs LPS

The influence of 3,3′,4,5′-TMS and 3,4′,5-TMS on intracellular O_2_^•−^ production in LPS-induced RAW 264.7 cells was assessed by DHE staining using flow cytometry. The time-dependent effect of LPS treatment (0.5, 1, 2, 4, 8 and 12 h) was tested by DHE staining and maximum induction was observed at 4 h (Fig. 8a); and thus this time point at 4 h was chosen for the following experiment. Treating with 3,3′,4,5′-TMS alone slightly decreased the O_2_^•−^ production at high concentration (50 µM, 4 h) while 3,4′,5-TMS showed no effect (Fig. 8b). Likewise, the cells were pretreated with 3,3′,4,5′-TMS and 3,4′,5-TMS (10, 50 µM) for 4 h, followed by co-treatment with LPS (1 µg/mL) for another 4 h. The results indicated that 3,3′,4,5′-TMS at the concentration of 50 µM moderately suppressed O_2_^•−^ production in LPS-stimulated RAW 264.7 macrophages (Fig. 8c, d) but no significant inhibition was observed in 3,4′,5-TMS treatment (Fig. 8e, f). On the other hand, pretreatment of 3,3′,4,5′-TMS and 3,4′,5-TMS both decreased the intracellular H_2_O_2_ level as determined by another fluorescence dye CM-H_2_DCFDA in RAW 264.7 cells stimulated with LPS (Fig. 9a, b). Exposure to LPS triggered the accumulation of MDA which were also reversed by 3,3′,4,5′-TMS and 3,4′,5-TMS (Fig. 9c). Taken together, both 3,3′,4,5′-TMS and 3,4′,5-TMS had the potential to alleviate oxidative stress induced by LPS.

## Discussion

In the present study by using LPS-induced RAW 264.7 macrophages, we showed the inhibitory effects and the underlying mechanisms of 3,3′,4,5′-TMS and 3,4′,5-TMS, two methoxy derivatives of resveratrol, on inflammatory pathogenesis. Our results strongly suggested that 3,3′,4,5′-TMS and 3,4′,5-TMS could suppress LPS-induced inflammation through inactivation of MAPK and NF-κB signaling pathways in RAW 264.7 cells. In addition, 3,3′,4,5′-TMS exhibited more potent effects against inflammation and oxidative stress than 3,4′,5-TMS.

Inflammation is involved in the development of various deadly illness such as arthritis, arteriosclerosis, cancer, liver diseases, neurological disorder, and renal disorders [20]. Macrophages function to control and clear infections, remove dead cells and derbies, and promote wound healing and tissue repair; nevertheless, prolonged and excessive activation of macrophages contributes to tissue damage and pathology in inflammatory diseases [21]. Upon stimulation of LPS in macrophages, upregulated expression of iNOS results in the burst of NO generation and contributes to the development of inflammation. Cyclooxygenase (COX) with COX-2 as the inducible isoform catalyzes the conversion from arachidonic acid (AA) to prostaglandin E2 (PGE2) which is also a pivotal indicator of inflammation [22]. Cytokines play important roles in regulating an inflammation process and various cells express different cytokines. IL-6 and TNF-α are the most common cytokines expressed in macrophages [23]. The present study showed that 3,3′,4,5′-TMS and 3,4′,5-TMS, two methoxy derivatives of resveratrol, alleviated inflammation through suppressing the secretions of NO, IL-6 and TNF-α and expressions of iNOS and COX-2 in LPS-induced RAW 264.7 cells. These results were in consistent with the previous study reporting that 3,4′,5-TMS possesses anti-inflammatory effect [19]. However, that earlier study only excludes the role of heme oxygenase-1 (HO-1) and the underlying molecular mechanisms remain to be explored. Of note, we were the first to suggest the anti-inflammatory activity of 3,3′,4,5′-TMS.

Our results strongly supported the protective effects of 3,3′,4,5′-TMS and 3,4′,5-TMS against inflammation in LPS-treated macrophages. Next, we examined the underlying mechanism. It has been well established that LPS activates TLR4 and triggers the downstream MAPKs and NF-κB signaling pathways, modulating inflammatory responses in RAW 264.7 cells [24]. JNK, p38, and ERK are kinase modules of MAPK family and participate in many pathological processes such as inflammation, cell apoptosis, gene transcription, and differentiation [5, 25, 26]. Normally, NF-κB is bound with IκB so that the complex cannot translocate into the nucleus from cytoplasm and maintain in an inactive form. Once the cells being exposed to extracellular stimuli like LPS, rapid phosphorylation by IKK, ubiquitination, and proteolytic degradation will happen to IκB, releasing NF-κB to translocate to the nucleus [27]. As a result, the transcription of a mass of pro-inflammatory cytokines and mediators including IL-6, TNF-α, and COX-2 was triggered [7]. Here we found that 3,3′,4,5′-TMS reduced the phosphorylation levels of JNK and p38 in MAPK pathway as well as the phosphorylation levels of IKKα/β and IκBα in NF-κB pathway. Similarly, 3,4′,5-TMS reduced the phosphorylation of all these proteins: JNK, p38, ERK, IKKα/β, IκBα, and p65. Besides, both 3,3′,4,5′-TMS and 3,4′,5-TMS exerted inhibition effect on the nuclear translocation of NF-κB p65. These results indicated that the anti-inflammatory effects of these two resveratrol derivatives are at least partially mediated through inhibiting MAPK and NF-κB activation. Contradiction about the impact of LPS on the expression of IκBα was found in the previous studies. Some studies showed that LPS treatment effectively suppressed the expression of IκBα [28, 29]; whilst other studies showed that LPS increased the phosphorylation of IκBα without affecting the expression of total protein [30, 31]. This different result might be caused by the difference in treatment time and/or source of antibodies. Our present results showed that LPS treatment induced the phosphorylation of IκBα which was reversed by 3,3′,4,5′-TMS and 3,4′,5-TMS but the expression of total IκBα was unchanged among different groups.

Extensive evidence supported the diverse beneficial effects of resveratrol against various diseases such as cancer, neurological disorders, diabetes, cardiovascular diseases and so on [32]. Regarding the remarkable therapeutic potentials of resveratrol, studies on the derivatives of resveratrol with more potent protective effects have raised attention. Resveratrol is widely known to activate Sirtuin 1 (SIRT1). SIRT1, a NAD+-dependent class III histone deacetylase, has been proved to take part in many pathophysiological processes including anti-inflammation via modulating specific proinflammatory mediators [33–35]. Furthermore, the deacetylation of NF-κB regulated by SIRT1 can suppress the expression of downstream signaling pathways, thus alleviate inflammation induced by LPS [22, 36, 37]. Resveratrol is reported to regulate MAPK and NF-κB signaling pathways for its immunomodulating functions [38]. Our recent study confirmed that resveratrol upregulated SIRT1 expression to ameliorate vascular dysfunction associated with diabetes and obesity [39]. Both 3,3′,4,5′-TMS and 3,4′,5-TMS are methoxy derivatives of resveratrol, sharing the similar structure with resveratrol, exhibit the same anti-inflammatory properties via suppressing MAPK and NF-κB signaling pathways. Importantly, 3,3′,4,5′-TMS and 3,4′,5-TMS showed differential effects that they did not upregulate SIRT1 expression.

It is well known that LPS can promote the production of ROS initially, and thereby ROS activate various signaling pathways to induce macrophage over-activation [40, 41]. We measured the time-dependent effect of LPS on intracellular O_2_^•−^ level indicated by DHE fluorescence and confirmed that LPS significantly stimulated ROS production at 4 h. The elevated O_2_^•−^ level was moderately suppressed by 3,3′,4,5′-TMS but was not altered by 3,4′,5-TMS; nevertheless, the overproduction of H_2_O_2_ as indicated by another fluorescence probe CM-H_2_DCFDA was suppressed by 3,3′,4,5′-TMS and 3,4′,5-TMS. DHE and CM-H_2_DCFDA are two most commonly applied fluorescence probes to measure ROS production. DHE is oxidized to red fluorescent ethidium by O_2_^•−^ whereas CM-H_2_DCFDA is oxidized to dichlorofluorescein (DCF) by H_2_O_2_. Different from the most specific fluorescent probe for superoxide detection of DHE, the range of ROS detected by CM-H_2_DCFDA is much broader [42]. This explains why 3,4′,5-TMS showed inhibitory effect in CM-H_2_DCFDA staining but not in DHE staining. MDA is a widely used and convenient biomarker for lipid peroxidation due to its facile reaction with thiobarbituric acid (TBA) [43]. The measurement of MDA content well reflects the oxidative stress level in the cells. The results showed that LPS-induced accumulation of MDA was reduced by the two compounds. Taking together, both 3,3′,4,5′-TMS and 3,4′,5-TMS could protect cells from oxidative stress. Previous studies on 3,3′,4,5′-TMS are very limited. Our results demonstrated that 3,3′,4,5′-TMS with one more methoxy group might exert more potent anti-inflammatory (as reflected by the NO release) and anti-oxidative properties (as shown by the fluorescence intensity and MDA content) than 3,4′,5-TMS.

Previous study demonstrated that resveratrol inhibits LPS-induced inflammation in macrophages through suppressing TLR4-NFκB/MAPKs signaling cascades [44]. Our present results also showed that the two resveratrol derivatives 3,3′,4,5′-TMS and 3,4′,5-TMS suppressed NFκB/MAPKs signaling pathways. Sharing similar structure with resveratrol, 3,3′,4,5′-TMS and 3,4′,5-TMS likely target TLR4, cell-surface receptor for LPS. However, this possibility of targeting TLR4 remains to be explored. Other resveratrol derivatives also possess anti-inflammatory effects, as for example, pterostilbene, 3,4,5,4′-tetramethoxystilbene, and 3,3′,4,5′-tetrahydroxystilbene, mediated through different signaling cascades including NFκB and/or MAPKs pathways [8]. The SAR among the resveratrol derivatives should be evaluated in future studies.

## Conclusions

In summary, our data revealed that 3,3′,4,5′-TMS and 3,4′,5-TMS suppress inflammatory responses in LPS-induced RAW 264.7 cells through the inhibition of MAPK and NF-κB pathways. Further in vivo studies on these two methoxy derivatives of resveratrol are warranted to develop them into therapeutic applications for inflammatory disorders considering that 3,3′,4,5′-TMS and 3,4′,5-TMS have great potential for treatment of inflammation.

## Data Availability

Not applicable.

## Abbreviations

3,3’,4,5’-TMS: 3,3’,4,5’-Tetramethoxy-*trans*-stilbene
3,4’,5-TMS: 3,4’,5-Trimethoxy-*trans*-stilbene
COX-2: Cyclooxygenase-2
DHE: Dihydroethidium
ERK: Extracellular signal-regulated kinase
IκB: Inhibitor of NF-κB
IKK: IκB kinase
IL-6: Interleukin-6
iNOS: Inducible nitric oxide synthase
JNK/SAPK: c-Jun N-terminal kinase/ stress-activated protein kinase
LPS: Lipopolysaccharide
MAPK: Mitogen-activated protein kinase
MDA: Malondialdehyde
MTT: 3-(4,5-Dimethylthiazol2-yl)-2,5-diphenyltetrazolium bromide
NF-κB: Nuclear factor kappa-light chain-enhancer of activated B cells
NO: Nitric oxide
SAR: Structure-activity relationship
TBA: Thiobarbituric acid
TNF-α: Tumor necrosis factor-α
